# Supplementary material for: Lithium Salt Catalyzed Ring-Opening Polymerized Solid-State Electrolyte with Comparable Ionic Conductivity and Better Interface Compatibility for Li-Ion Batteries
Source: Membranes (Basel). 2022 Mar 16;12(3):330. doi: 10.3390/membranes12030330 (PMC8955661; doi:10.3390/membranes12030330)
Supplement: Supplementary file 1 [file membranes-12-00330-s001.zip › membranes-1610879-supplementary.pdf]

membranes

Supplementary Materials

Lithium Salt Catalyzed Ring-Opening Polymerized Solid-State Electrolyte with Comparable Ionic Conductivity and Better Interface Compatibility for Li-Ion Batteries

Wei Zhang [1], Sujin Yoon [1], Lei Jin [1], Hyunmin Lim [1], Minhyuk Jeon [1], Hohyoun Jang [1], Faiz Ahmed [2] and Whangi Kim [1,*]

[1] Department of Applied Chemistry, Konkuk University, Chungju 27478, Korea; arno_zw@hotmail.com (W.Z.); ysj920126@naver.com (S.Y.); jinlei8761@naver.com (L.J.); tree3367@naver.com (H.L.); jeonminh97@naver.com (M.J.); 201417450@kku.ac.kr (H.J.)
[2] Grenoble INP, LEPMI, University of Grenoble Alpes, 38000 Grenoble, France; faiz2310@gmail.com
* Correspondence: wgkim@kku.ac.kr

Citation: Zhang, W.; Yoon, S.; Jin, L.; Lim, H.; Jeon, M.; Jang, H.; Ahmed, F. Lithium Salt Catalyzed Ring-Opening Polymerized Solid-State Electrolyte with Comparable Ionic Conductivity and Better Interface Compatibility for Li-Ion Batteries. *Membranes* **2022**, *12*, 330. https://doi.org/10.3390/membranes12030330

Academic Editor: Giovanni Battista Appetecchi

Received: 9 February 2022
Accepted: 10 March 2022
Published: 16 March 2022

Publisher's Note: MDPI stays neutral with regard to jurisdictional claims in published maps and institutional affiliations.

Copyright: © 2022 by the authors. Licensee MDPI, Basel, Switzerland. This article is an open access article distributed under the terms and conditions of the Creative Commons Attribution (CC BY) license (https://creativecommons.org/licenses/by/4.0/).

Table S1. The explanation of main abbreviation words and used in this article.

Abbreviation	Full Name
LiFSI	lithium bis(fluorosulfonyl)imide
FTIR	Fourier transform infrared
^{1}H-NMR	Proton nuclear magnetic resonance
FE-SEM	Field emission scanning electron microscopy
EIS	Electrochemical impedance spectroscopy
LSV	Linear sweep voltammetry
GPEs	Gel polymer electrolytes
SPEs	Solid-state polymer electrolytes
CROP	Cationic ring-opening polymerization
EOM	3-ethyl-3-oxetanemethanol
CV	Cyclic voltametric
t_{Li}	Li-ion transference number
ESW	Electrochemical stability window
CD	charge-discharge
Csp	discharge specific capacity
η	coulombic efficiency

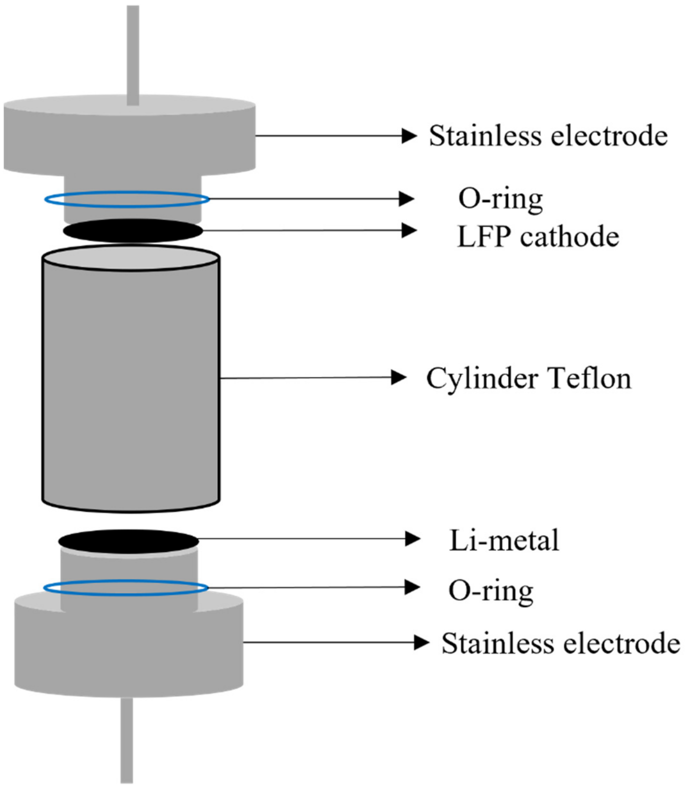

Figure S1. Configuration of symmetry Swagelok cell.

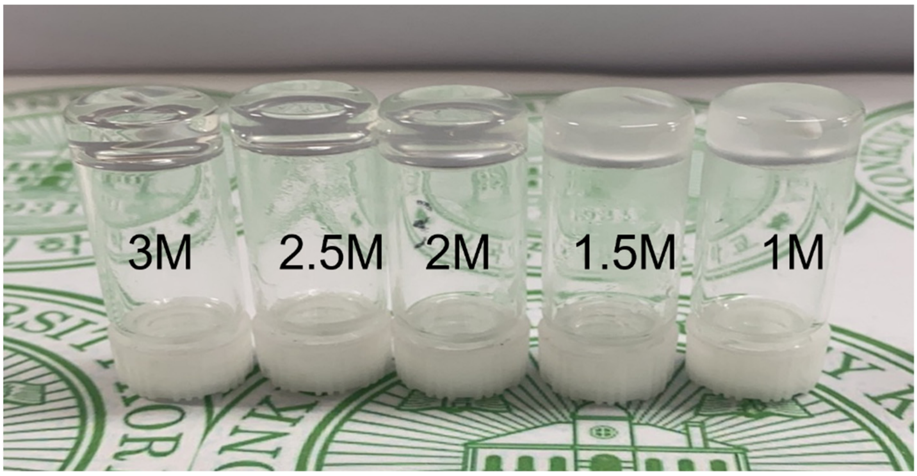

Figure S2. Photographing of varied concentrated LiFSI with EOM after 54 hrs.

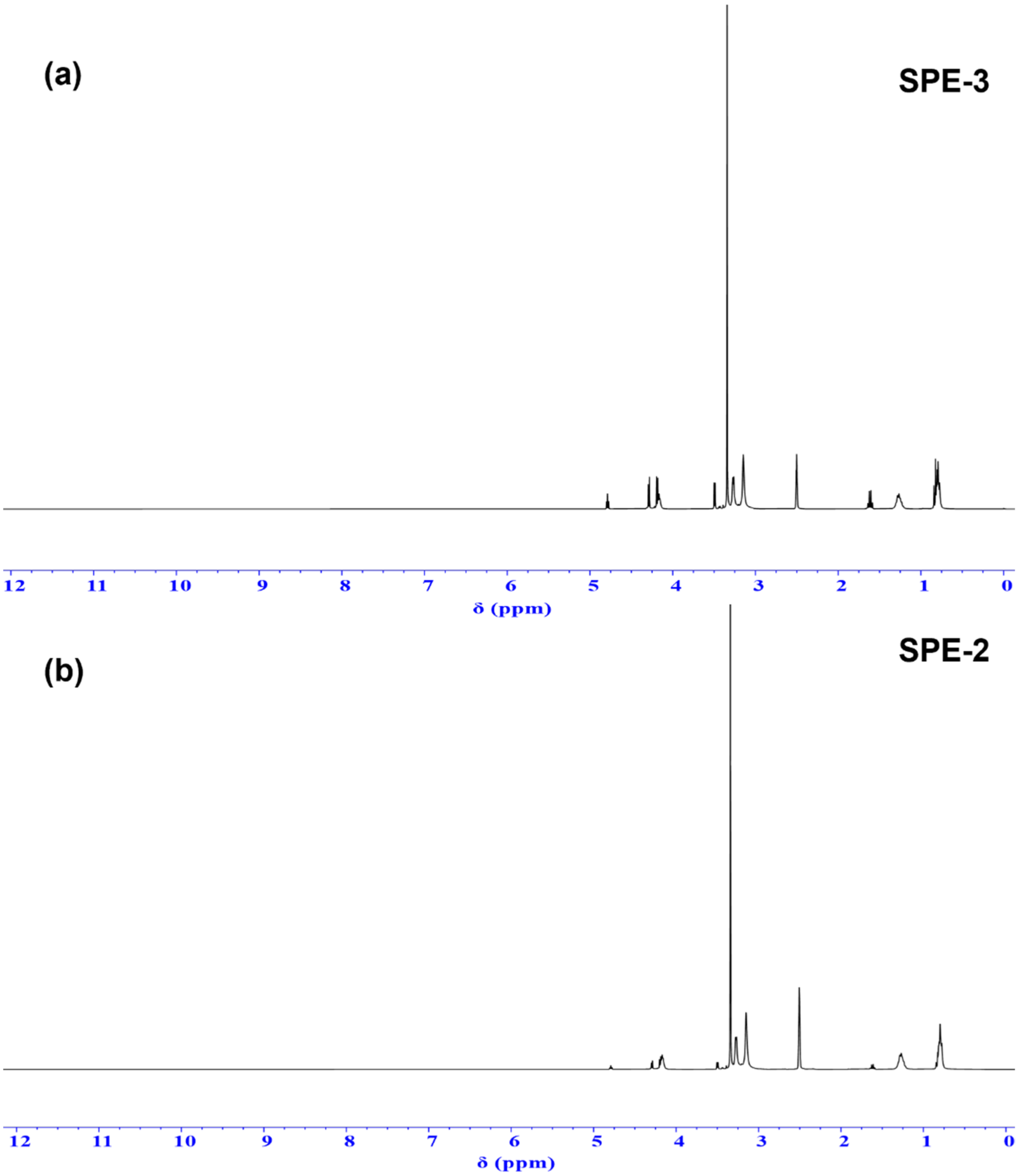

Figure S3. ^{1}H-NMR spectrum of SPE-3(a) and SPE-2(b).

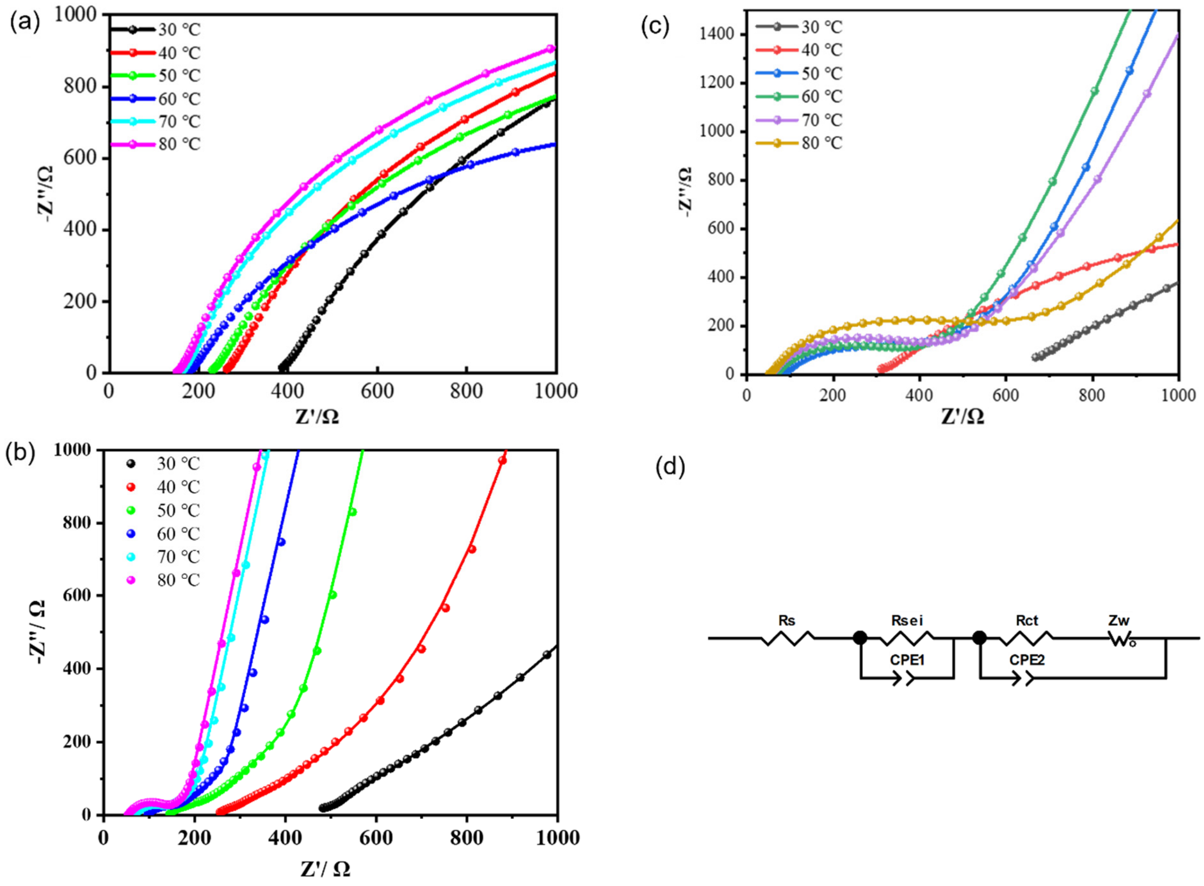

Figure S4. Nyquist curves of SPE-2(**a**), SPE-2.5(**b**) with fitting plots, and SPE-3(**c**); equivalent circuit (**d**).

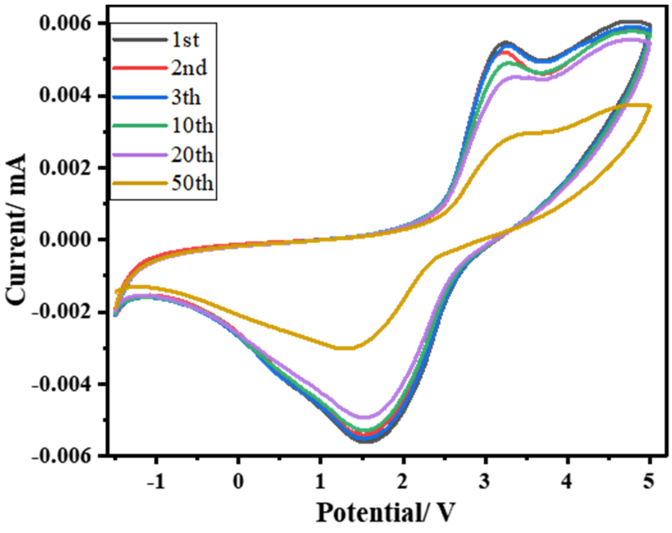

Figure S5. CV sweeping 50 cycles of SPE-2.5 electrolyte with the dummy cell over the potential range from −1.5 to 5V, scan rate at 25mv/s.

Table S2. Analytical parameters for the calculation of t_{Li+} of SPE-2.5.

ΔU (V)	I_0 (μA)	I_s (μA)	R_0 (Ω)	R_s (Ω)	t_{Li+}
0.01	2.92	2.13	160	225	0.75

Table S3. Comparison of properties of polymer electrolyte reported based on ring-opening polymerization.

Components	Salt/Pasticizer/Solvent	σ (mS/cm) [a]	t_{Li^+} [b]	Stability vs (Li^+/Li) (V) and Cathode	Ref.
PEO	LiTFSI/no/ACN	0.0004	0.4	4.8	[1]
DGEPEG, PEGDA	LiTFSI/no/no	0.053	N/A	4.7, LFP	[2]
POSS, P(EO-co-PO)	LiTFSI/ no/THF	0.11	0.62	5.4, LFP	[3]
GLYMO, EDGE	LiTFSI/no/Ethanol	0.026	0.37	4.9, LTO	[4]
GLYMO, DGEPEG	LiClO4/no/no	0.12	N/A	N/A	[5]
SPE-2.5	LiFSI/no/no	0.45	0.75	3.75, LFP	This work

[a] at 25 °C

[b] All lithium cation transference numbers (t_{Li^+}) reported in Table S2 were measured using the Bruce-Vincent method.

acetonitrile (ACN), tetrahydrofuran (THF), lithium bis(trimethanesulfonyl)imide (LiTFSI), LiFePO4-LFP, $Ni_{1/3}Mn_{1/3}Co_{1/3}O_2$ -NMC, $Li_4Ti_5O_{12}$(LTO), Poly (ethylene oxide) (PEO), Diglycidylether of polyethylene glycol (DGEPEG), Poly (ethylene glycol) diacrylate (PEGDA), Polyhedral oligomeric silsequioxane (POSS), Poly (ethylene-co-propylene oxide) (P (EO-co-PO)), (3-glycidyloxypropyl) trimethoxy silane (GLYMO), Ethyl glycol diglycidyl ether (EDGE),.

References

1. Cheng, H.; Zhu, C.; Huang, B.; Lu, M.; Yang, Y. Synthesis and electrochemical characterization of PEO-based polymer electrolytes with room temperature ionic liquids. *Electrochim. Acta* **2007**, *52*, 5789–5794. doi: 10.1016/j.electacta.2007.02.062.
2. Duan, H.; Yin, Y.X.; Zeng, X.X.; Li, J.Y.; Shi, J.L.; Shi, Y.; Wen, R.; Guo, Y.G.; Wan, L.J. In situ plasticized polymer electrolyte with double-network for flexible solid-state lithium-metal batteries. *Energy Storage Mater.* **2018**, *10*, 85–91. doi: 10.1016/j.ensm.2017.06.017.
3. Hsu, S.T.; Tran, B.T.; Subramani, R.; Nguyen, H.T.T.; Rajamani, A.; Lee, M.Y.; Hou, S.S.; Lee, Y.L.; Teng, H. Free-standing polymer electrolyte for all-solid-state lithium batteries operated at room temperature. *J. Power Sources* **2020**, *449*, 227518. doi: 10.1016/j.jpowsour.2019.227518.
4. Vélez, J.F.; Aparicio, M.; Mosa, J. Covalent silica-PEO-LiTFSI hybrid solid electrolytes via sol-gel for Li-ion battery applications. *Electrochim. Acta* **2016**, *213*, 831–841. doi: 10.1016/j.electacta.2016.07.146.
5. Popall, M.; Andrei, M.; Kappel, J.; Kron, J.; Olma, K.; Olsowski, B. ORMOCERs as inorganic-organic electrolytes for new solid state lithium batteries and supercapacitors. *Electrochim. Acta* **1998**, *43*, 1155–1161. doi: 10.1016/S0013-4686(97)10014-7.
